# The chromatin reader protein ING5 is required for normal hematopoietic cell numbers in the fetal liver

**DOI:** 10.3389/fimmu.2023.1119750

**Published:** 2023-05-18

**Authors:** Sophia Y.Y. Mah, Hannah K. Vanyai, Yuqing Yang, Anne K. Voss, Tim Thomas

**Affiliations:** ^1^ Epigenetics and Development Division, Walter and Eliza Hall Institute of Medical Research, Melbourne, VIC, Australia; ^2^ Department of Medical Biology, University of Melbourne, Melbourne, VIC, Australia

**Keywords:** ING5, chromatin, KAT6A, KAT7, fetal, hematopoiesis

## Abstract

ING5 is a component of KAT6A and KAT7 histone lysine acetylation protein complexes. ING5 contains a PHD domain that binds to histone H3 lysine 4 when it is trimethylated, and so functions as a ‘reader’ and adaptor protein. KAT6A and KAT7 function are critical for normal hematopoiesis. To examine the function of ING5 in hematopoiesis, we generated a null allele of *Ing5*. Mice lacking ING5 during development had decreased foetal liver cellularity, decreased numbers of hematopoietic stem cells and perturbed erythropoiesis compared to wild-type control mice. *Ing5^–/–^* pups had hypoplastic spleens. Competitive transplantation experiments using foetal liver hematopoietic cells showed that there was no defect in long-term repopulating capacity of stem cells lacking ING5, suggesting that the defects during the foetal stage were not cell intrinsic. Together, these results suggest that ING5 function is dispensable for normal hematopoiesis but may be required for timely foetal hematopoiesis in a cell-extrinsic manner.

## Introduction

The mammalian inhibitor of growth (ING) family consists of five proteins, ING1 to ING5, defined through sequence homology (1, 2). These proteins are chromatin ‘reader’ proteins as they all contain a plant homeodomain (PHD) finger (3), which is prominent in chromatin adaptor proteins (4). ING proteins are involved in the regulation of a wide range of critical cellular processes including DNA repair, apoptosis, cell cycle and epigenetic regulation of gene expression *via* association with histone acetyltransferases (HATs) or histone deacetylases (HDACs) (5, 6).

The PHD fingers of all mammalian ING proteins and yeast orthologues bind to mono-, di- or tri-methylated lysine 4 of histone H3 (H3K4me1/2/3) (7). The strongest binding occurs with H3K4me3. The affinity decreases 10-fold with the removal of successive methyl groups (8–17). This binding is specific to H3K4 methylation, as histone H3 methylated at other lysine residues (lysine 9) or histone H4 mono-, di- or trimethylated at lysine 20 (H4K20me1/2/3) is not bound (3, 8). H3K4me3 is enriched in the promoter regions and immediately downstream of transcription start sites of active genes and is associated with gene activation (7, 18). Dysregulation of ING proteins is associated with aberrant gene expression in cancers (19–22).

ING5 has been detected in distinct protein complexes, which contain a MYST (MOZ, Ybf2/Sas3, Sas2 and Tip60) protein family enzyme subunit. The MYST proteins are a family of histone acetyltransferases with diverse roles in chromatin regulation (23–25). ING5 was found in KAT6A (formerly known as MOZ, monocytic leukaemia zinc finger gene), KAT6B (formerly MORF or Querkopf) and KAT7 (formerly HBO1) complexes (26). Both KAT6A and KAT7 have essential roles in regulating the hematopoietic system (27–30). KAT6A is essential for the formation of definitive hematopoietic stem cells (HSCs) during embryonic development (27), and the maintenance of HSC in adult bone marrow (28). The role of KAT6A in hematopoiesis is dependent on its acetyltransferase activity (31). These findings suggest that how the acetyltransferase activity is directed to chromatin is important for the function of these proteins. KAT6A is required for pre-B cell proliferation in germinal centres and for the maturation of CD8+ T cells (32–34). KAT7 also has essential roles in the hematopoietic system, and like KAT6A, KAT7 is critically important for maintenance of HSCs (29), as well as in lineage commitment, in particular erythropoiesis (35) and T cell development (36). KAT7 function is also critical in thymic epithelial cells for clonal selection of T cells in the thymus (37).

Chromosomal translocations involving the *KAT6A* gene (24, 38, 39) or the *KAT6B* gene (40–42) cause aggressive forms of acute myeloid leukaemia. KAT7 dysregulation is associated with a variety of cancers (43) and, similar to *KAT6A*, *KAT7* chromosomal translocations cause leukaemia (44). Both KAT6A and KAT7 are promising targets for anti-cancer therapy (45–47), in particular leukaemia, and a drug inhibiting the enzymatic activity of KAT6A is in clinical trials.

Since the complexes containing the MYST family proteins KAT6A and KAT7 have essential roles in hematopoiesis, and both contain the adaptor protein ING5 we undertook a study to examine the role of ING5 in hematopoiesis.

## Materials and methods

### Animal strains and alleles

**Figure 1:**
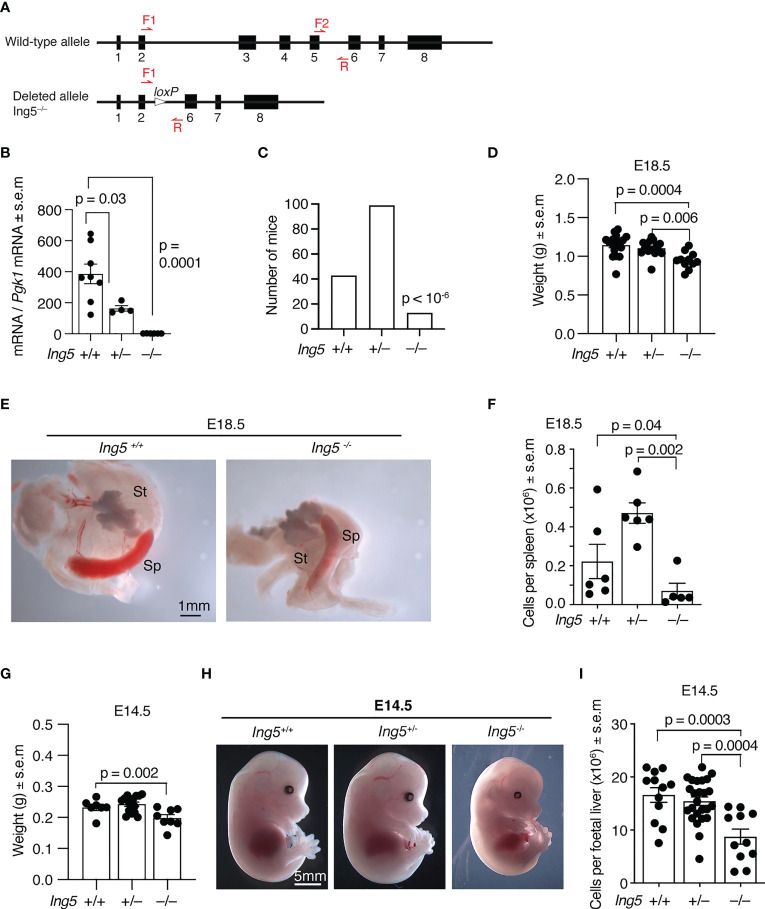
Gross phenotypic effects of loss of ING5. **(A)** The wild-type and germline null allele of the *Ing5* gene used in this study. Exons are numbered. Red arrows indicate position of oligonucleotide primers used for genotyping. *LoxP*, Cre-recombinase target sequence. Details of the targeting strategy and the intermediate conditional allele are displayed in **Supplementary Figure 1**. **(B)** Levels of *Ing5* mRNA quantified by RT-qPCR in whole E10.5 *Ing5^–/–^*, *Ing5^+/–^* and *Ing5^+/+^* embryos. N = 6 *Ing5^–/–^*, 4 *Ing5^+/–^* and 8 *Ing5^+/+^* embryos. **(C)** Numbers of *Ing5^–/–^*, *Ing5^+/–^* and *Ing5^+/+^* mice generated by mating *Ing5^+/–^* x *Ing5^+/–^* mice at 3 weeks of age (weaning). A total of 155 mice were genotyped. *Ing5^–/–^* were underrepresented at weaning (p < 10^-6^). **(D)** Body weight of E18.5 foetuses. N= 11 *Ing5^–/–^*, 15 *Ing5^+/–^* and 17 *Ing5^+/+^* foetuses. **(E)** Representative images of *Ing5^–/–^* and *Ing5^+/+^* E18.5 spleens. St, stomach; Sp, spleen. **(F)** Quantification of the number of cells in the spleen at E18.5 using an automated hematology analyser. **(G)** Body weights of E14.5 foetuses. N = 8 *Ing5^–/–^*, 16 *Ing5^+/–^* and 7 *Ing5^+/+^* foetuses. **(H)** Representative images of *Ing5^–/–^*, *Ing5^+/–^* and *Ing5^+/+^* foetuses at E14.5. **(I)** Foetal liver cellularity at E14.5. The number of nucleated hematopoietic cells was quantified using an automated hematology analyser. N = 11 *Ing5^–/–^*, 25 *Ing5^+/–^* and 12 *Ing5^+/+^* foetuses. Each dot represents one foetal liver. Data are displayed as mean ± s.e.m. and were analysed by one-way ANOVA followed by Tukey’s multiple comparisons test **(B, D, F,G, I)**, or are displayed as absolute numbers and were analysed by cumulative binomial probability analysis **(C)**. Each dot represents one animal **(B, D, F, G, I)**.

Mice with *loxP* sites flanking exons 3 to 5 of the *Ing5* gene (*Ing5^fl^*) were generated by OZgene Pty Ltd, Bently, Western Australia, using Bruce 4 ES cells, which are C57BL/6 derived. Removal of exons 3 to 5 was achieved by crossing the *Ing5^fl^* mice to a *Cre-*deleter mouse strain (48), which produced a frame shift and a premature stop codon and generated the germline deleted *Ing5^–^* allele used in this study. The region of deletion (**Supplementary Figure 1**; **Figure 1A**) encodes part of the N-terminal ING domain and the nuclear localisation signal. Mice carrying the *Ing5^–^* allele were backcrossed to wild-type C57BL/6J mice for more than 8 generations. Mice were genotyped by PCR using a common forward oligonucleotide 1 (TGCTGGGACTGTTTACAAATTAGA) together with a reverse oligonucleotide 2 (AAAGGAGTGAACAATACAGCATGA) detecting the wild-type (322 bp product) allele or a reverse oligonucleotide (ATGTACCGAATGTGGGAACTAAAT) detecting the null allele (528 bp product) in the same reaction. Quantitative reverse transcriptase PCR using a pair of oligonucleotides amplifying cDNA 3 prime of the deleted exons (forward CCAGAAGCCTGAGTGTCTCC and reverse TGCCAGTCTGTTGATGAAGC) was performed on RNA isolated from E10.5 embryos using Qiagen RNeasy Mini Kit (Qiagen 217004) followed by cDNA synthesis using Super Script III Reverse Transcriptase (Invitrogen 18080085) and RT-qPCR amplification using SYBR Hi-ROX (Bioline QT605-05). Values for RT-qPCR are displayed as arbitrary units relative to the housekeeping gene *Pgk1*.

### Tissue collection

Foetal livers were isolated from E14.5 embryos, and cells were dissociated by passing through a 40 μm cell strainer (Corning 352340). Bone marrow, thymus and spleen cells were isolated from transplant recipients using standard techniques. Peripheral blood and tissue cell counts were obtained using an automated hematology analyser (ADVIA 120; Siemens).

### Hematopoietic transplant experiments

All irradiated recipients were CD45.1^+^ C57BL/6 female mice aged 6-8 weeks. Mice received irradiation (2 x 5.5 Gy 3 h apart) and were given drinking water supplemented with 10 mg/ml of neomycin (Sigma N6386) for 3 weeks post-irradiation. Competitive transplantation experiments were conducted using 1x10^6^ bone marrow cells from femurs of CD45.1^+^ mice (competitor cells) combined with 0.5 x 10^6^ cells of CD45.2^+^ E14.5 foetal livers (donor). Cells were injected into the tail vein of irradiated CD45.1^+^ host mice. Three hosts were used per donor. Peripheral blood was isolated at 4 weeks and 16 weeks post-transplantation for analyses. Haematopoietic organs were recovered at 16 weeks post-transplantation.

### Flow cytometry

For hematopoietic organs, equal numbers of cells were stained with primary antibodies (**Supplementary Table 1**) using standard procedures. Cell types were identified using cell surface markers as shown in **Supplementary Table 2**. Expression of CD34 and CD135 were used to identify a stem cell population, designated LT-HSCs, and progenitor cell populations, as previously published (49–51) or, alternatively, SLAM markers CD150 and CD48 (52, 53) were used to identify a stem cell population designated HSCs and progenitor cell populations. After excess secondary antibodies were removed by washing with FACS buffer, all pelleted cells were resuspended in Fluoro-Gold viability dye (8 μg/ml; Sigma 39286). Samples were then analysed on flow cytometry analysers, LSRIIW, LSRIIC, or Fortessa1 (BD Biosciences). Data were analysed using FlowJo v10.4 (Treestar). Representative gating strategies are displayed in **Supplementary Figures 2-7**.

### Statistical analysis

Data are presented as means ± s.e.m. and were analysed using a graphing and statistics software (Prism 9 version 9.4.1, GraphPad; or R version 4.2.2, The R Foundation for Statistical Computing Platform). The specific statistical tests used, and number of observations are stated in the figure legends.

## Results

To examine the role of ING5 in hematopoiesis we generated a mouse strain in which exons 3, 4 and 5 were deleted in the germline (**Supplementary Figure 1**; **Figure 1A**). Exons 3 to 5 encode part of the conserved N-terminal ING domain and the nuclear localisation signal. Splicing around exons 3 to 5 results in a frameshift obliterating the PHD finger. Therefore, no functional protein is produced from the mutated locus. Quantitative reverse transcriptase PCR was used to assess *Ing5* mRNA levels in whole E10.5 embryos from heterozygous intercross matings. No *Ing5* RNA was detected in *Ing5^–/–^* embryos using primers that amplify mRNA encoded 3’ of the genomic deletion, showing that the mutation targets the *Ing5* locus and that any splicing out of frame results in degradation of the out-of-frame mRNA (**Figure 1B**).

Using this allele on an inbred C57BL/6 background, we studied the effect of loss of ING5 by generating homozygous embryos from heterozygous intercross matings. Only a third of the number of *Ing5^–/–^* mice expected were present at weaning (p < 10^-6^, **Figure 1C**) with some mice dying soon after birth. At E18.5 before birth, *Ing5^–/–^* foetuses were externally morphologically normal, although smaller than wild-type and *Ing5^+/–^* foetuses (p = 0.0004 and 0.006; **Figure 1D**). Noteworthy was a reduction in spleen cellularity in E18.5 *Ing5^–/–^* foetuses compared to wild-type and *Ing5^+/–^* littermate controls (p = 0.04 and 0.002; **Figures 1E, F**), which resembled, but was not as significant as the loss of spleen cellularity in *Kat6a^–/–^* (*Moz^–/–^*) foetuses (27). A moderate growth retardation was visible at E14.5 in *Ing5^–/–^* foetuses compared to wild-type littermate controls (p = 0.002; **Figures 1G, H**). A reduction in foetal liver cellularity to 53% of wild-type was apparent in E14.5 *Ing5^–/–^* foetuses (p = 0.0003; **Figure 1I**). This reduction in foetal liver cells was still apparent when the moderate growth retardation was taken into consideration (62% of wild-type; p = 0.005; data not shown).

**Figure 2:**
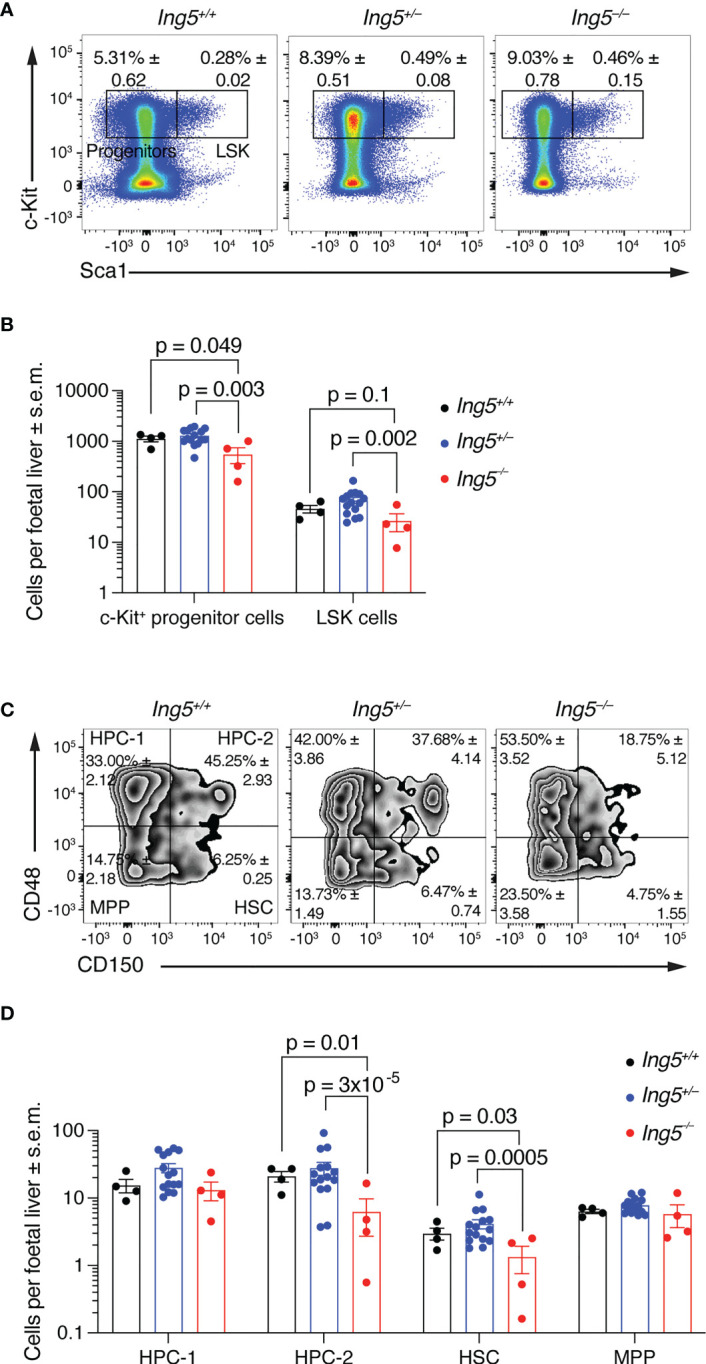
Loss of ING5 causes a reduction in HSC and progenitor numbers. E14.5 foetal liver hematopoietic cell suspensions were analysed for HSC and progenitors within the LSK compartment using SLAM markers CD48 and CD150. Gating strategy as shown in **Supplementary Figure 2**. **(A)** Representative flow cytometry plots for each genotype gated on live cells. Numbers within plots are average percentage ± s.e.m. of total live cells. **(B)** Bar graph showing the number of LSK and c-Kit^+^ progenitor cells per foetal liver. **(C)** Representative flow cytometry plots for identification of stem and progenitor cells using CD150 and CD48 gated on the LSK compartment. Numbers within plots are average percentage ± s.e.m. of total LSK cells. **(D)** Bar graph showing the number of stem and progenitor populations per foetal liver. N = 4 *Ing5^–/–^*, 15 *Ing5^+/–^* and 4 *Ing5^+/+^* foetuses. Data are displayed as mean ± s.e.m. and were analysed after log transformation by two-way ANOVA with genotype and cell type as the independent factors followed by Šídák’s multiple comparisons test. Each dot represents data from one animal. HPC-1, hematopoietic progenitor cells 1; HPC-2, hematopoietic progenitor cells 2; HSC, hematopoietic stem cells; MPP, multipotent progenitor cells. Detailed gating strategy in **Supplementary Figure 2**. **Supplementary Table 1** displays antibodies used, and **Supplementary Table 2** cell surface markers used for various hematopoietic cell populations.

To determine the role of ING5 in foetal liver hematopoiesis, we examined the hematopoietic stem cell compartment at E14.5 using flow cytometry (**Supplementary Figure 2**). The number of lineage negative (Lin^neg^) c-Kit positive (c-Kit^+^) progenitor cells per foetal liver was significantly reduced in *Ing5^–/–^* foetuses compared to wild-type and *Ing5^+/–^* littermate controls (p = 0.049 and 0.003; **Figures 2A, B**). *Ing5^–/–^* foetuses also showed a tendency of a reduction in Lin^neg^, c-Kit^+^, Sca-1^+^ (LSK) cells compared to wild-type and *Ing5^+/–^* littermate controls (p = 0.1 and 0.002; **Figures 2A, B**). Examining the stem cell compartment further by subdividing the LSK population based on the expression of CD48 and CD150 (52, 53), we found a significant decrease in the hematopoietic progenitor cell 2 (HPC-2) population (p = 0.01 and 3x10^-5^; HPC-2; **Figures 2C, D**) and the HSC populations in *Ing5^–/–^* foetuses compared to wild-type and *Ing5^+/–^* littermate controls (p = 0.03 and 0.005; **Figures 2C, D**).

**Figure 3:**
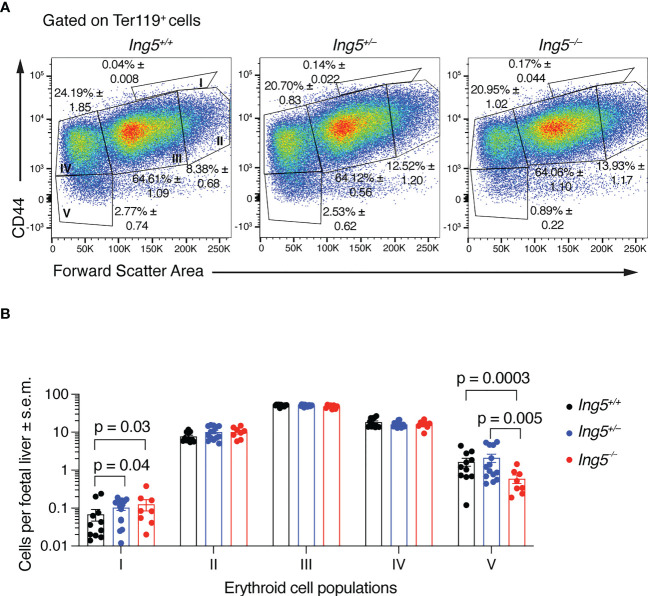
Loss of ING5 results in an increase in proerythroblasts and a decrease in the mature erythrocyte population. **(A)** Representative flow cytometry plots for each genotype, gated on Ter-119+ cells. Population I, proerythroblasts; II, basophilic erythroblasts; III, polychromatic erythroblasts; IV, orthochromatic erythroblasts; IV, orthochromatic erythroblasts and reticulocytes; V, mature red blood cells. Numbers within plots are average percentage ± s.e.m. of the total Mac-1^neg^ Gr-1^neg^ CD45.2^neg^ Ter-119^+^ cells. **(B)** Bar graphs of the number cells in each sub-population of erythroblasts per foetal liver. N = 8 *Ing5^–/–^*, 14 *Ing5^+/–^* and 11 *Ing5^+/+^* foetuses. Data are displayed as mean ± s.e.m. and were analysed after log transformation by two-way ANOVA with genotype and cell type as the independent factors followed by Šídák’s multiple comparisons test. Each dot represents number of cells of one animal. Gating strategy in **Supplementary Figure 3**.

Together, the results presented in the previous section suggested that the principal effect of loss of ING5 was on the stem and progenitor cell populations. In E14.5 foetal livers, the majority of progenitors are required to generate definitive erythroid cells in large numbers to support the growth of the embryo. Therefore, we examined erythropoiesis using flow cytometry (54) (**Supplementary Figure 3**). This analysis showed that there was an accumulation of proerythroblasts (population I) in *Ing5^–/–^* and *Ing5^+/–^* foetuses compared to wild-type littermate controls (p = 0.03 and 0.04; **Figures 3A, B**) but not in basophilic (II), polychromatic (III) or orthochromatic erythroblasts and reticulocytes (IV; **Figures 3A, B**). Interestingly, *Ing5^–/–^* foetuses displayed a significant reduction in mature erythrocytes compared to wild-type and *Ing5^+/–^* littermate controls (p = 0.0003 and 0.005; **Figures 3A, B**). Therefore, loss of ING5 resulted in a reduction in progression through the proerythroblasts stage of erythropoiesis and a reduction in mature erythrocytes at E14.5, which were mild enough to allow development of the *Ing5^–/–^* foetuses to term.

**Figure 4:**
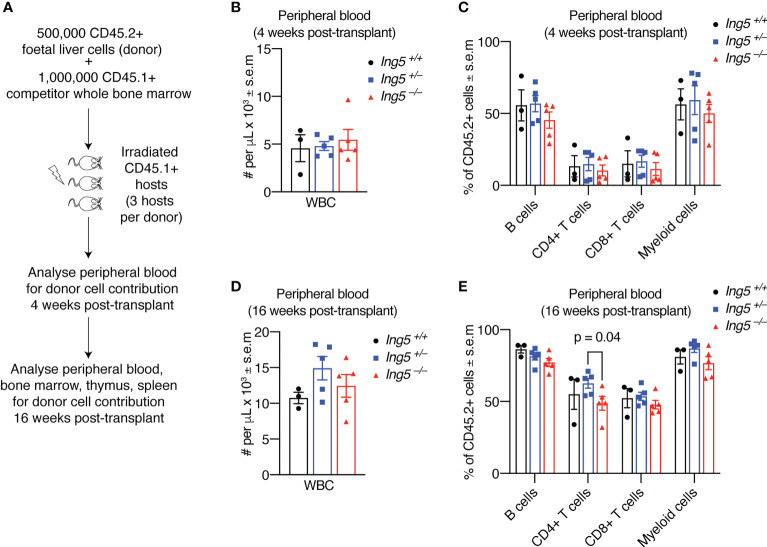
Loss of ING5 does not have a major effect on the contribution of donor cells to mature cell types in the peripheral blood after short-term or long-term reconstitution. Analysis of peripheral blood by flow cytometry 4 weeks and 16 weeks after competitive foetal liver cell transplantation. **(A)** Protocol for competitive transplantation of foetal liver cells into irradiated recipient mice and subsequent analyses. **(B)** Quantification of nucleated white blood cells in the peripheral blood using an automated hematology analyser 4 weeks after transplantation. **(C)** Proportion of CD45.2^+^ donor cells among mature cell types in the peripheral blood 4 weeks post-transplantation. **(D)** Quantification of nucleated white blood cells in the peripheral blood using an automated hematology 16 weeks after transplantation. **(E)** Proportion of CD45.2^+^ donor cells among mature cell types in the peripheral blood at 16 weeks post-transplantation. N = 5 *Ing5^–/–^*, 5 *Ing5^+/–^* and 3 *Ing5^+/+^* foetal liver samples, transplanted into 3 recipients each. Each dot represents the average of three recipient mice that received cells from the same donor. Data are displayed as mean ± s.e.m. and were analysed by one-way ANOVA followed by Tukey’s multiple comparisons test. Gating strategy in **Supplementary Figure 4**.

To further examine the function of ING5 in hematopoiesis we performed competitive foetal liver hematopoietic cell transplants. We transplanted 500,000 E14.5 foetal liver test cells mixed with 1,000,000 adult bone marrow competitor cells. A minimum of three individual foetal livers (biological replicates) for each genotype were transplanted, each into three recipients (technical replicates) making a total of at least nine transplants for each genotype studied. The technical replicates for each biological replicate were averaged. The foetal liver cells had a CD45.2 cell surface phenotype and both the recipient and competitor cells had a CD45.1 cell surface phenotype (**Figure 4A**). Examination of the peripheral blood (**Supplementary Figure 4**) at 4 weeks post-transplantation (**Figures 4B, C**) or at 16 weeks post-transplantation (**Figures 4D, E**) showed no significant differences in the number of white blood cells or the proportion of leukocyte populations, apart from a slight increase in CD4+ T cells in the *Ing^+/–^* sample compared to the *Ing5^–/–^* (**Figure 4E**).

**Figure 5:**
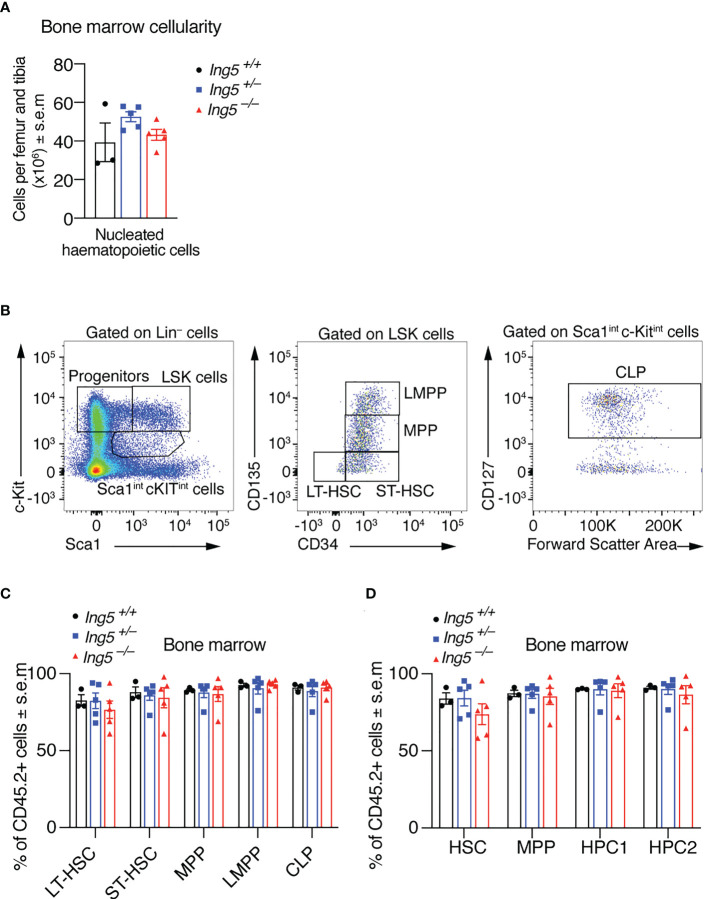
Loss of ING5 does affect donor cell contribution to the production of HSCs and progenitor cells in the bone marrow after competitive foetal liver cell transplantation. Analysis of the bone marrow 16 weeks after competitive foetal liver cell transplantation. **(A)** Quantification of nucleated hematopoietic cells in the bone marrow of recipients using an automated hematology analyser. **(B)** Gating strategy to identify stem and progenitor subsets using CD135, CD34 and CD127. **(C)** Proportion of CD45.2^+^ donor cell contribution to each major subtype of HSC and progenitor cells identified using CD34, CD135 and CD127. **(D)** Proportion of CD45.2^+^ donor cell contribution to each major subtype of HSC and progenitor cells identified using CD48 and CD150. N = 5 *Ing5^–/–^*, 5 *Ing5^+/–^* and 3 *Ing5^+/+^* foetal liver samples in **(A, C, D)**. Data are displayed as mean ± s.e.m. and were analysed by one-way ANOVA followed by Tukey’s multiple comparisons test. Each dot represents the average of three recipients that received cells from the same donor. Detailed gating strategy in **Supplementary Figures 2**; **5**.

**Figure 6:**
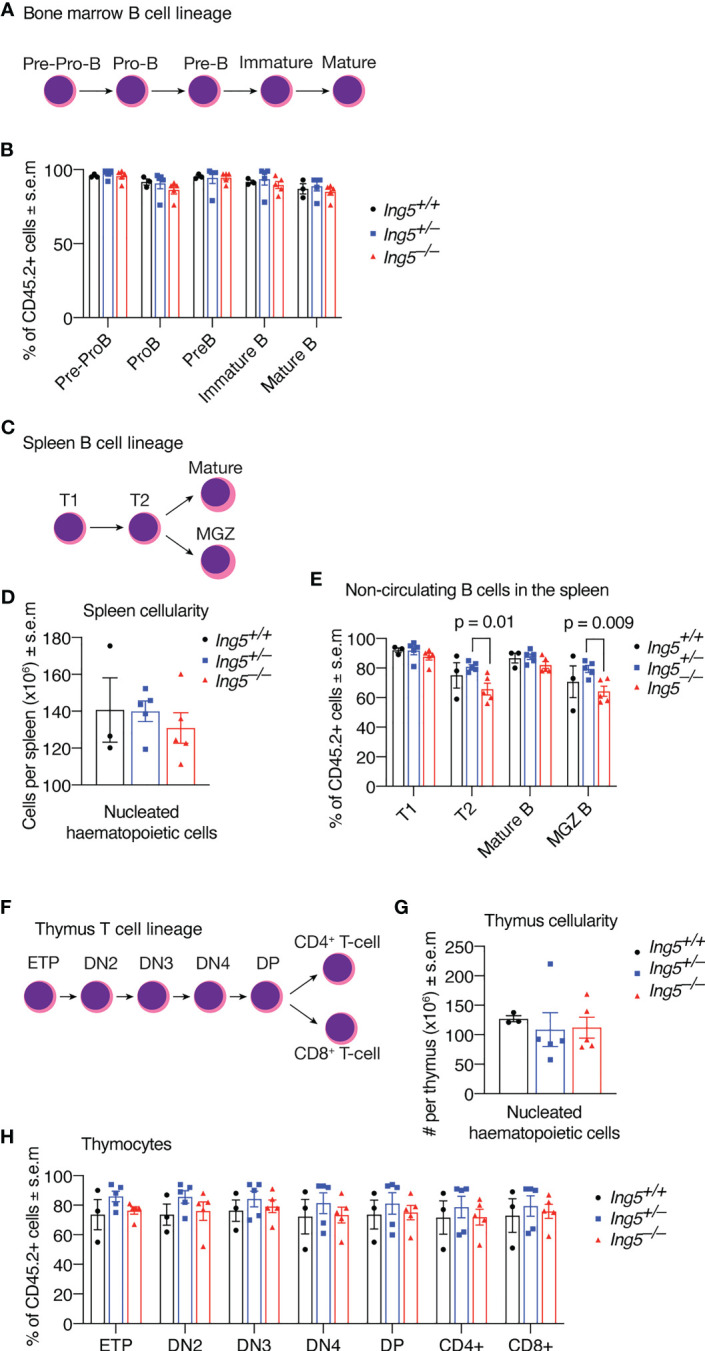
Loss of ING5 does not have a major effect on donor cell contribution to lymphoid development in bone marrow, spleen and thymus after competitive foetal liver cell transplantation. Analysis of bone marrow, spleen and thymus cells by flow cytometry 16 weeks after competitive foetal liver cell transplantation. **(A)** Major subtypes of B cell progenitors in the bone marrow. **(B)** Proportion of CD45.2^+^ donor cell contribution to each major subtype of B cell progenitors and mature B cells in the bone marrow. **(C)** Major subtypes of B cells in the spleen. **(D)** Quantification of nucleated hematopoietic cells in the spleen. **(E)** Proportion of CD45.2^+^ donor cell contribution to each major subtype of B cell progenitors and mature B cells in the spleen. **(F)** Major subtypes of T cells in the thymus. **(G)** Quantification of nucleated hematopoietic cells in the thymus. **(H)** Proportion of CD45.2^+^ donor cells contribution to each major subtype of T cells progenitors and mature cell types. N = 5 *Ing5^–/–^*, 5 *Ing5^+/–^* and 3 *Ing5^+/+^* foetal liver samples transplanted into 3 recipients each. Each dot represents the average of the three recipients that received cells from the same donor. Data are displayed as mean ± s.e.m. and were analysed by one-way ANOVA followed by Tukey’s multiple comparisons test. MGZ B cells: marginal zone B cells; T1, transitional zone 1 B cells; T2, transitional zone 2 B cells. ETP, early thymic progenitors; DN2-4, double negative (CD4^neg^ CD8^neg^) cell stage 2-4; DP, double positive for CD4 and CD8.

After 16 weeks, we examined the stem cell compartment in the bone marrow of the transplanted mice studied in **Figure 4**, using the flow cytometric gating strategy shown in **Supplementary Figure 5**. Bone marrow cellularity was not significantly different between genotypes (**Figure 5A**). No significant differences between genotypes were seen in stem or early progenitor cell populations generated by the transplanted cells (**Figures 5B-D**) identified using two methods, namely by subdividing LSK cells based on CD135 and CD34 expression (**Figures 5B, C**; detailed gating strategy in **Supplementary Figure 5**) or CD150 and CD48 expression (**Figure 5D**; gating strategy in **Supplementary Figure 5**). Similarly, no significant differences in the contribution of donor cells of different genotypes to common lymphoid progenitors (CLPs) were detected (**Figures 5B, C**). The transplanted cells were distinguished from host or competitor cells by the CD45.2 cell surface phenotype. No major significant differences in the B cell linage were observed between recipients receiving *Ing5^–/–^* or *Ing5^+/+^* littermate control foetal liver cells when bone marrow and spleen were examined (**Figures 6A-E**; **Supplementary Figure 6**). A slight difference was seen between the proportion of T2 and marginal zone B cells in the spleen between *Ing5^–/–^* and *Ing5^+/–^*, but *Ing5^–/–^* and *Ing5^+/+^* donor cells were not significantly different (**Figure 6E**). Similarly, no significant differences in the T cell linage were observed between recipients receiving *Ing5^–/–^* or *Ing5^+/+^* littermate control foetal liver cells when the thymus was examined (**Figures 6F-H**; **Supplementary Figure 7**).

Overall these results show that while there are significant differences in the cellularity of the foetal liver and foetal stem and progenitor cells, these differences are not cell-intrinsic to the long-term repopulating stem cells.

## Discussion

In this study we observed that about two thirds of the *Ing5^–/–^* mice died between birth and weaning, presumably accounting for the deaths observed soon after birth. The surviving *Ing5^–/–^* mice were normal and fertile. We observed some abnormalities in hematopoiesis in *Ing5^–/–^* foetuses. Since ING5 is a member of the KAT6A and KAT7 chromatin regulatory complexes, this suggested that, like KAT6A and KAT7, ING5 may have a function in the hematopoietic stem cell compartment. At E18.5 the spleens of *Ing5^–/–^* foetuses were severely cytopenic. The spleen, a site of erythropoiesis during foetal development, is populated by HSCs coming from the foetal liver (55). This phenotype is similar to, although not as severe as the phenotype of foetuses lacking the histone acetyltransferase KAT6A (MOZ) (27). Similar to *Kat6a* heterozygous foetuses (27), foetuses lacking ING5 showed a reduction in the numbers of CD48^+^ CD150^+^ HSCs.

During development, the rapidly growing foetus requires the production of a large number of red blood cells. Examining erythropoiesis, we found an increase in the most immature erythroid progenitor cells and a reduction in the number of mature red blood cells. These findings are similar to the effect of a conditional deletion of *Kat7* or *Kat6a* on erythropoiesis in midgestation embryos (27, 29). Loss of either KAT7 or KAT6A causes a delay in erythropoiesis maturation resulting in the accumulation of early erythroid progenitors that does not prevent differentiation of mature definitive red blood cells (27, 29). In addition, disrupted erythropoiesis was also observed after shRNA knockdown of *Kat7* (*Hbo1* (35);.

In contrast to loss of KAT6A or KAT7 function, loss of ING5 function does not affect the ability of ING5 deficient cells to repopulate the hematopoietic system of a lethally irradiated recipient mouse. This suggests that, unlike the effects of loss of KAT6A or KAT7, the effects of loss of ING5 are not cell autonomous but affect the interaction of hematopoietic cells with surrounding cells, particularly in the foetal spleen. Alternatively, other aspects of foetal development affecting hematopoietic cells may affect foetal liver hematopoiesis, which might include a developmental delay.

The results described in this paper suggest that the ING5 adaptor protein is not required for many of the essential, cell-intrinsic functions of either KAT6A or KAT7 in the hematopoietic system. KAT6A and KAT7 are the enzyme subunits of their respective complexes, which, in addition to ING5, contain BRPF1-3 (KAT6A and KAT7) (26, 35, 56, 57) or JADE1-3 (KAT7) (26, 58). The BRPF and JADE family proteins are also adaptor proteins containing bromo and PHD domains and so direct their respective complexes to chromatin (59). Indeed, alternative splice variants of JADE may lack the ING5 binding domain, resulting in a KAT7 complex which does not contain ING5 and has a changed histone lysine specificity (11, 58). This suggests that ING5 has a specific role in modulating the activity of chromatin regulatory complexes in which it is found. KAT7 has a global function in regulating H3K14ac (46, 60–62), but under different conditions has been shown to acetylate H4 (26). KAT6A has a function in regulating H3K23ac (63) and H3K9ac at specific loci (64–67). H3K14ac and H3K23ac are the two most abundant histone acetylation modifications (68), suggesting that the complexes generating the modifications are present throughout the genome (62). However, there is an enrichment of at least H3K14ac and KAT7 at transcription start sites (62, 69). Transcription start sites are also enriched for H3K4me3 (7, 18), the modification bound by ING5 (3, 8). It is possible that the function of ING5 is related to the enrichment of these complexes at transcription start sites rather than at all genomic locations where KAT6A and KAT7 are found.

ING5 has an identical domain structure to ING4 and these domains are highly conserved (2). ING4 is an alternative subunit for the KAT7 complex (26) and has also been pulled-down in the KAT6A complex (70), suggesting that there might be a degree of redundancy between ING4 and ING5 in the KAT6A complex as well as in the KAT7 complex.

In conclusion we have shown that ING5 has a function in the hematopoietic system during foetal development. However, the loss of ING5 does not cause severe defects of the same magnitude as the complete loss of the function of the protein complexes in which it is found.

## Data availability statement

The original contributions presented in the study are included in the article/**Supplementary Materials**. Further inquiries can be directed to the corresponding authors.

## Ethics statement

The animal study was reviewed and approved by Walter and Eliza Hall Institute Ethics Committee.

## Author contributions

Contribution: SM, YY, HV conducted experiments and analysed data. TT and AV wrote the manuscript. TT and AV conceived and supervised the project. All authors contributed to the article and approved the submitted version.
