# Supplementary material for: The chromatin reader protein ING5 is required for normal hematopoietic cell numbers in the fetal liver
Source: Front Immunol. 2023 May 18;14:1119750. doi: 10.3389/fimmu.2023.1119750 (PMC10232820; doi:10.3389/fimmu.2023.1119750)
Supplement: Supplementary file 1 [file DataSheet_1.docx]

**The chromatin reader protein ING5 is required for normal haematopoietic cell numbers in the foetal liver**

Sophia YY Mah^1,2^, Hannah K Vanyai^1,2^, Yuqing Yang^1,2^, Anne K. Voss^1,2^ and Tim Thomas^1,2^

*^1^Walter and Eliza Hall Institute of Medical Research, Melbourne, Victoria 3052, Australia*

*^2^Department of Medical Biology, University of Melbourne, Melbourne, Victoria 3052, Australia*

**7 Supplementary Figures and 2 Supplementary Tables**

**
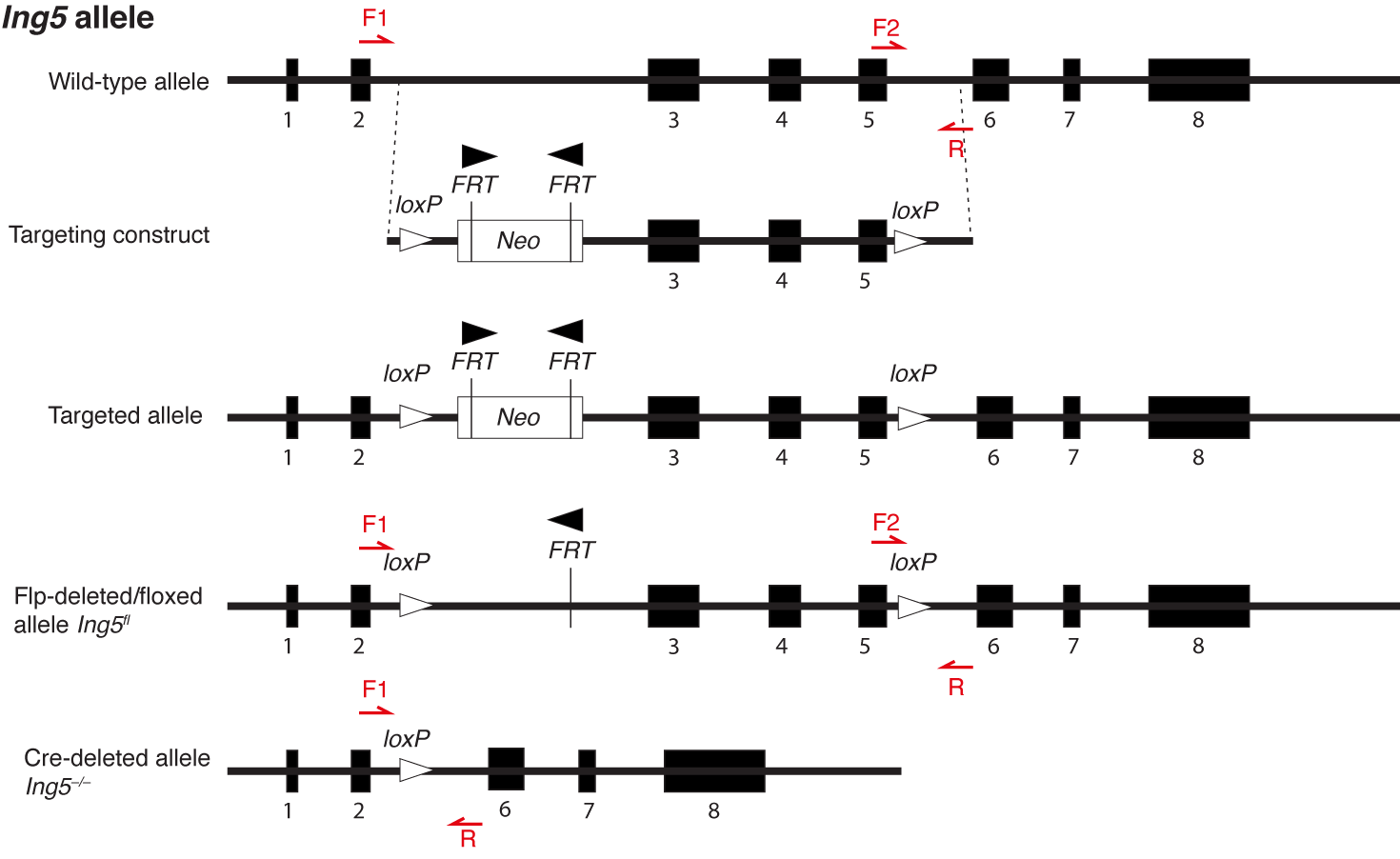
**

**Supplementary Figure 1. Schematic gene locus map showing the strategy for creating the null *Ing5* allele** (not to scale).

The exons of the *Ing5* gene are numbered, the exon/intron structure is indicated by blocks and lines. Red arrows indicate position of oligonucleotide primers used for genotyping. *FRT*, Flippase recognition target sequence. *LoxP*, Cre-recombinase target sequence. *Neo,* Neomycin phosphotransferase selectable marker gene. The *Ing5* wild-type (*Ing5^+/+^*) and the *Ing5* null allele (*Ing5^–^*) are also displayed in Figure 1A.

**
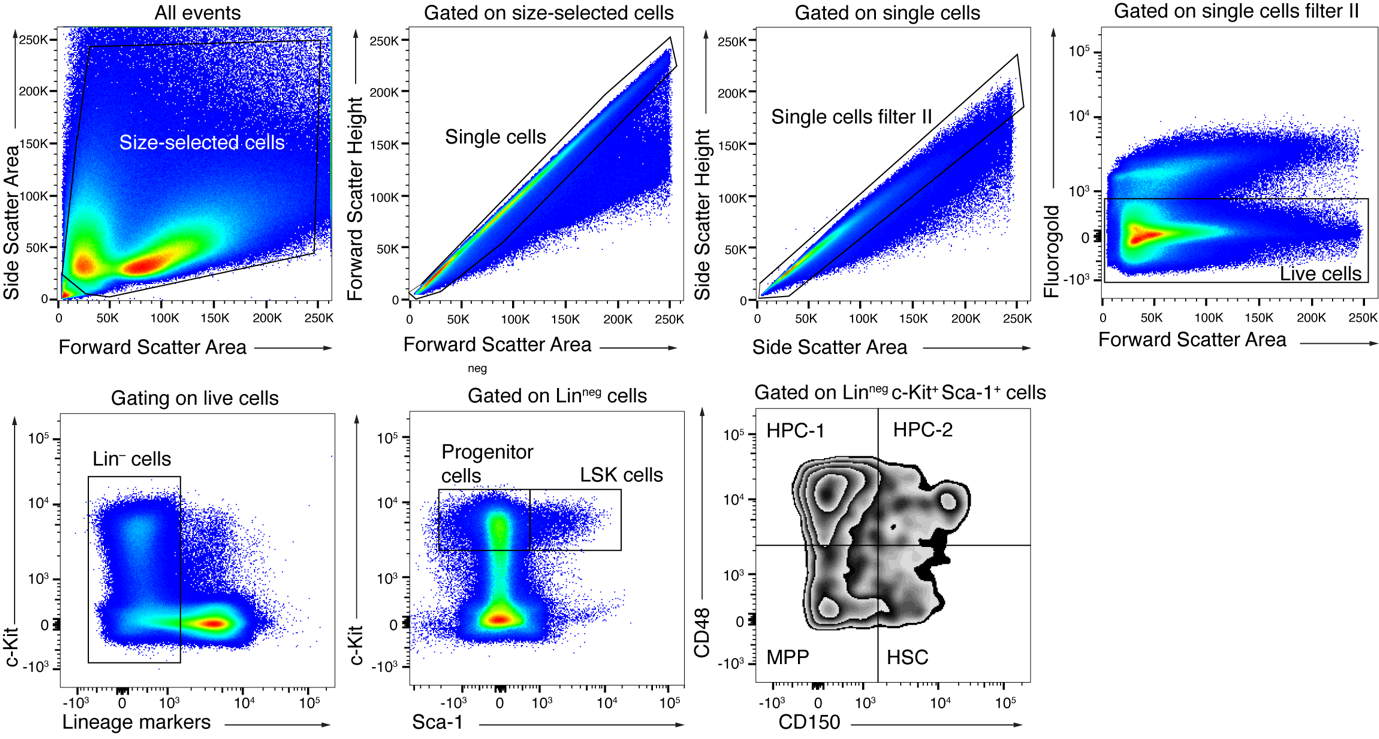
**

**Supplementary Figure 2. Representative flow cytometry plots showing the gating strategy for the identification of HSCs and progenitor cell populations using signalling and lymphocytic activation molecule (SLAM) family members CD48 and CD150.**

HPC-1, haematopoietic progenitor cells 1; HPC-2, haematopoietic progenitor cells 2; Lin, lineage markers used in foetal liver cell sorts (B220, CD19, Gr-1, CD71, Ter-119, CD4, CD8); MPP: multipotent progenitors. The cell surface marker protein combinations for specific cell types are listed in Supplementary Table 2.

**
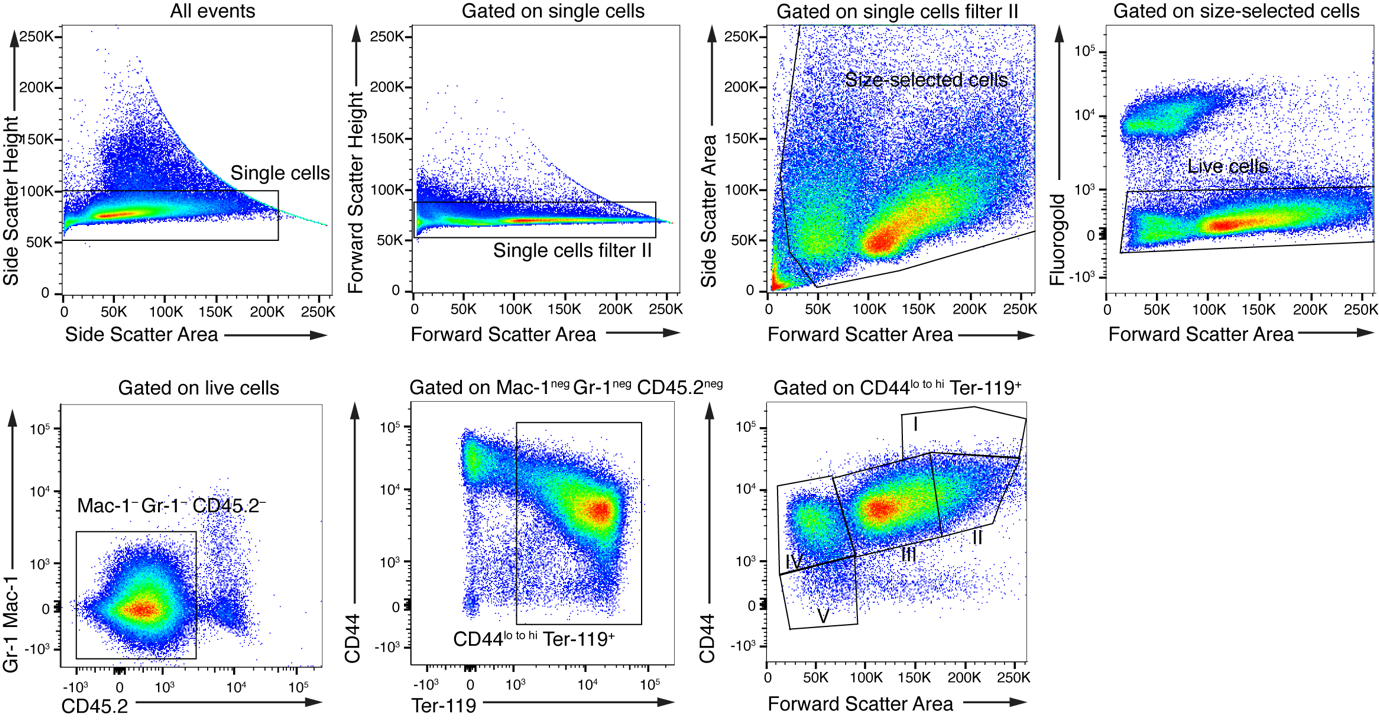
**

**Supplementary Figure 3. Representative flow cytometry plots showing the gating strategy for the identification of erythroid progenitors and mature red blood cells in the foetal liver.**

Population I, proerythroblasts; II, basophilic erythroblasts; III, polychromatic erythroblasts; IV, orthochromatic erythroblasts and reticulocytes; V, mature red blood cells. The cell surface marker protein combinations for specific cell types are listed in Supplementary Table 2.


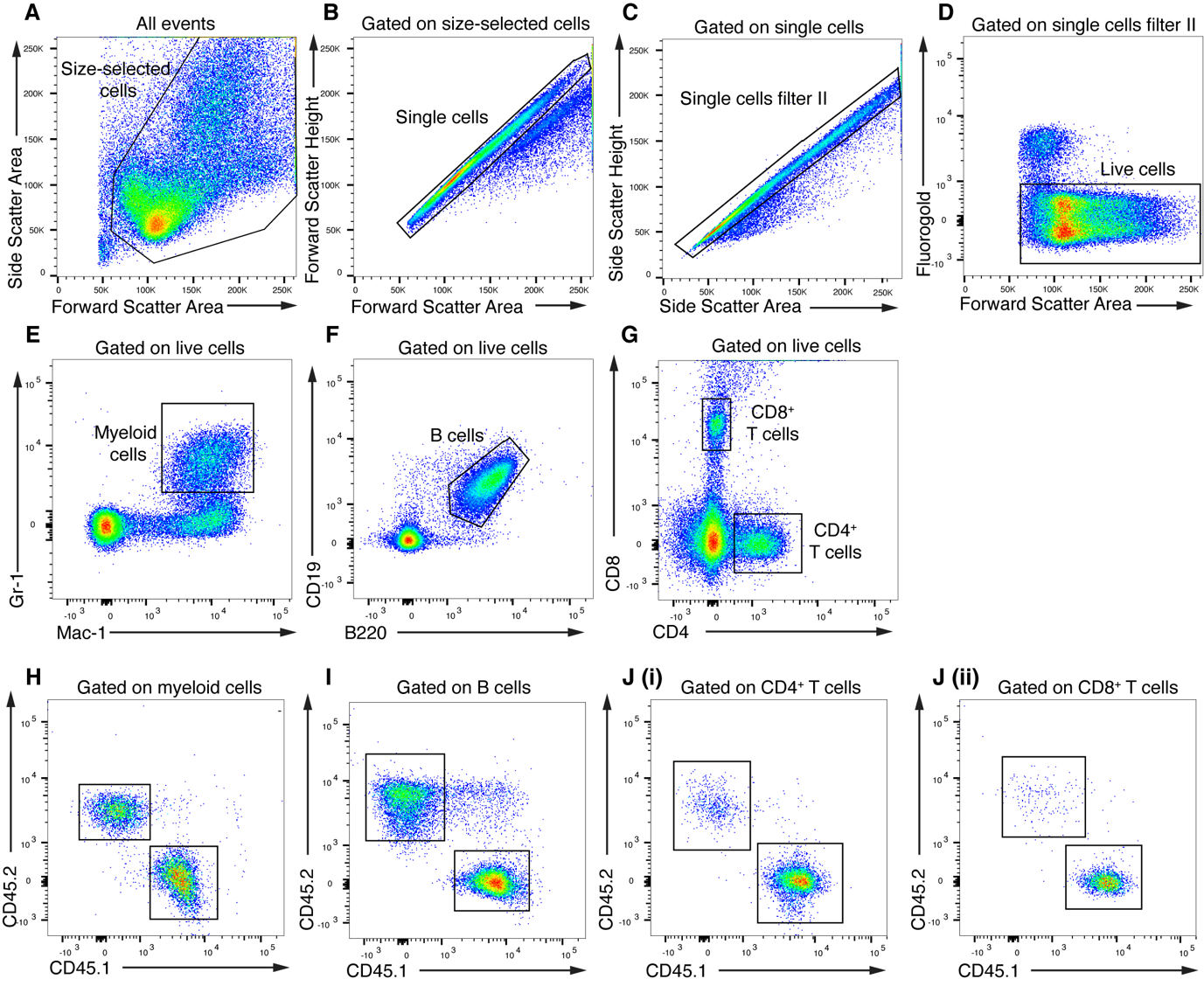


**Supplementary Figure 4. Representative flow cytometry plots showing the gating strategy for identification of major cell types in the peripheral blood of recipient mice after competitive foetal liver cell transplantation.**

Samples were stained for cell surface markers for B cells, T cells, and myeloid cells. Donor cells express CD45.2, whereas competitor and host cells express CD45.1. The cell surface marker protein combinations for specific cell types are listed in Supplementary Table 2.


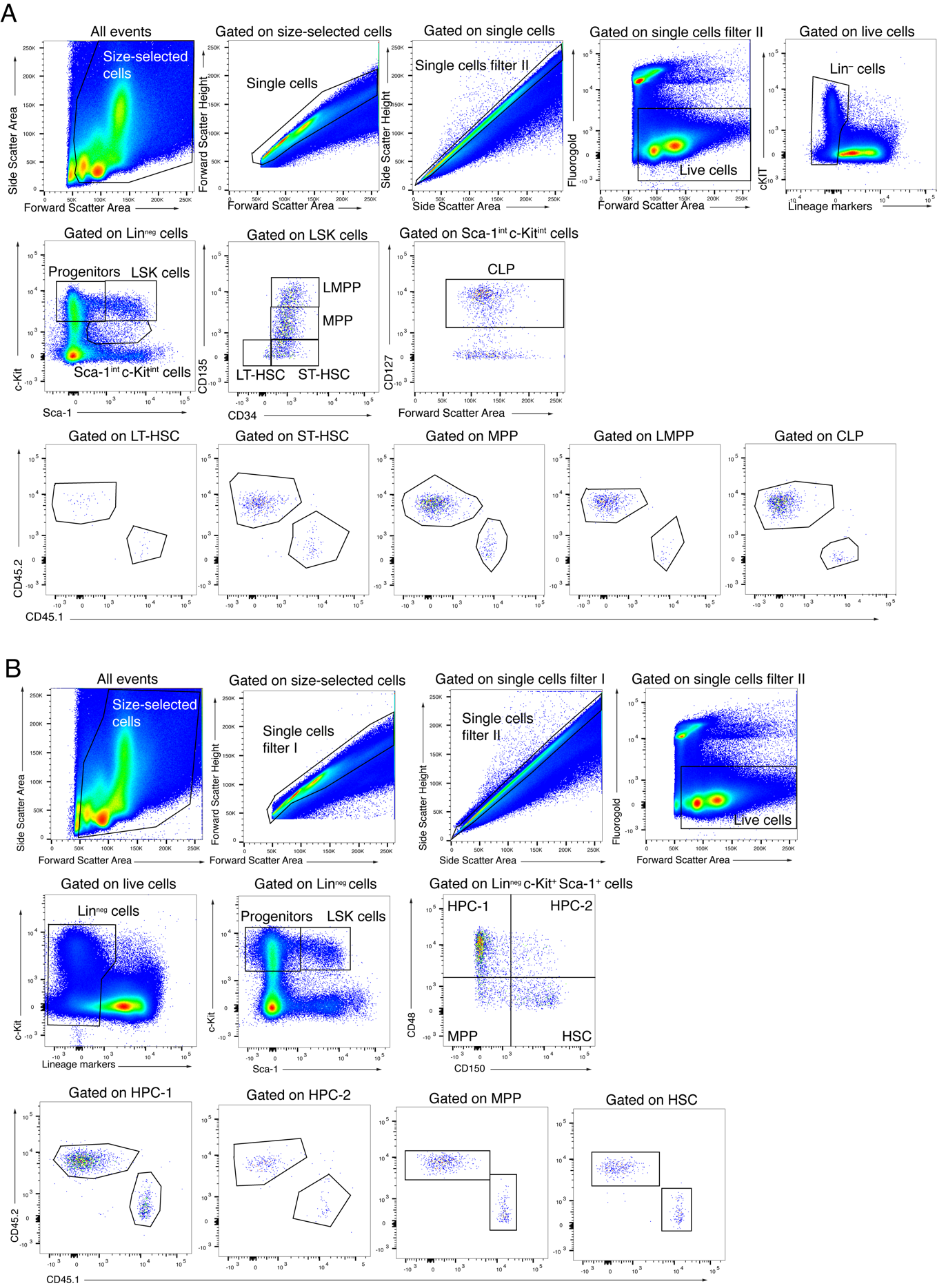


**Supplementary Figure 5.** **Representative flow cytometry plots showing two gating strategies for the detection of stem and progenitor cells in the bone marrow 16 weeks after competitive foetal liver cell transplantation.**

Donor cells expressed CD45.2, whereas competitor and host cells expressed CD45.1.

(A) Identification of stem and progenitor cell types, namely long-term repopulating HSCs (LT-HSCs), short-term repopulating HSCs (ST-HSCs), multipotent progenitor cells (MPPs), lymphoid-primed multipotential progenitor cells (LMPPs), and common lymphoid progenitor cells (CLPs) based on separation of LSK cells by CD34 and CD135 expression, as well as CD127 expression, as published (1,2,3).

(B) Identification of HSC subtypes, namely haematopoietic progenitor cells 1 (HPC-1), haematopoietic progenitor cells 2 (HPC-2), multipotent progenitor cells (MPPs) and HSCs, based on separation of LSK cells by signalling and lymphocytic activation molecule (SLAM) family member CD48 and CD150 expression (4,5).

Lin, lineage markers used for adult specimens (B220, CD19, Mac-1, Gr-1, CD71, Ter-119, CD4, CD8). The cell surface marker protein combinations for specific cell types are listed in Supplementary Table 2.

**
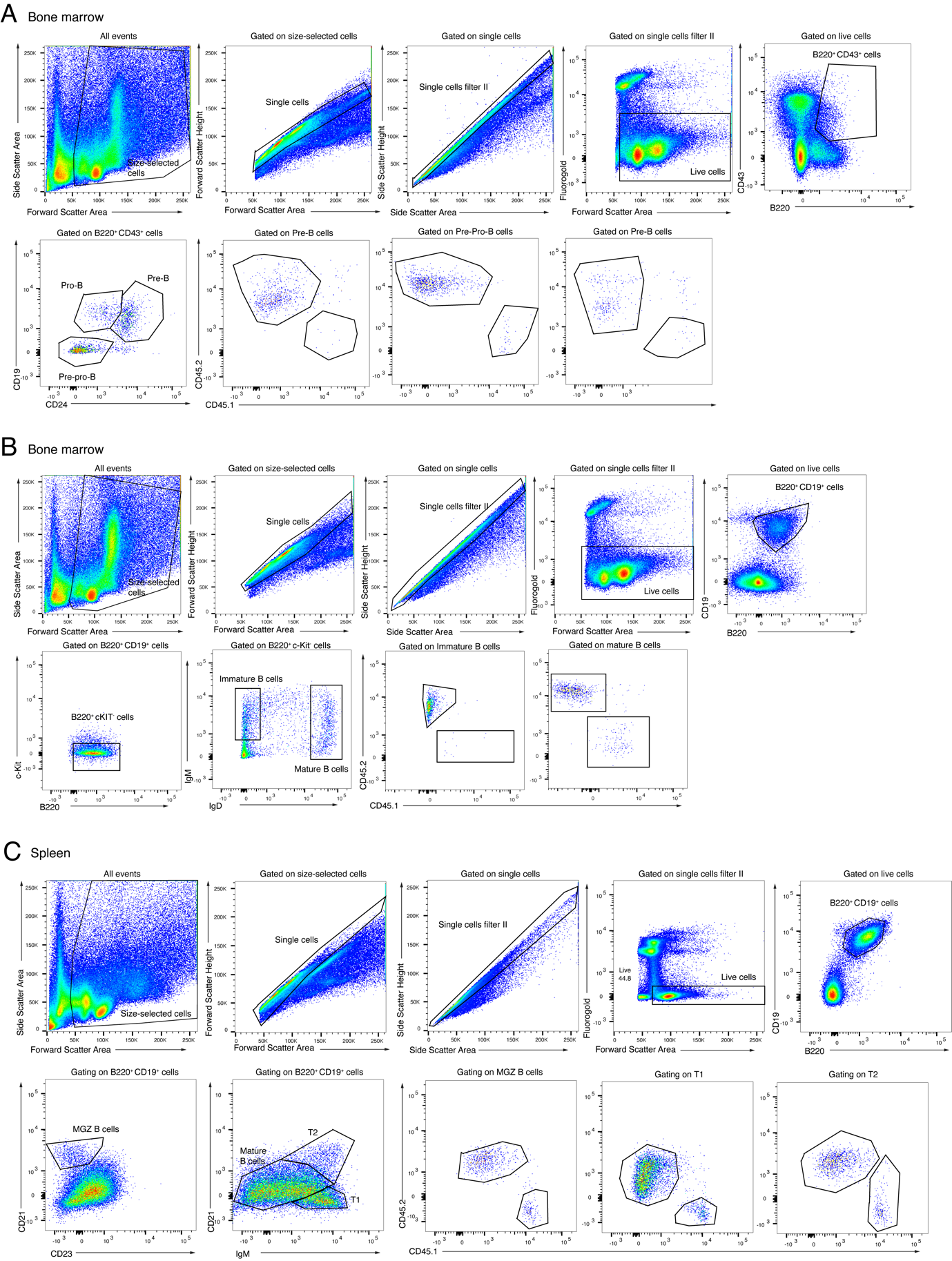
**

**Supplementary Figure 6. Gating strategy for the analysis of bone marrow, spleen and thymus 16 weeks after competitive foetal liver cell transplantation.**

Donor cells expressed CD45.2, whereas competitor and host cells expressed CD45.1.

(A) Representative flow cytometry plots showing the identification of B cell progenitors within the bone marrow.

(B) Representative flow cytometry plots showing the identification of mature and immature B cells in the bone marrow.

(C) Representative flow cytometry plots showing the identification transitional immature B cells and resident mature B cells in the spleen. MGZ B cells, marginal zone B cells; T1, transitional zone 1 B cells; T2, transitional zone 2 B cells.

The cell surface marker protein combinations for specific cell types are listed in Supplementary Table 2.

**
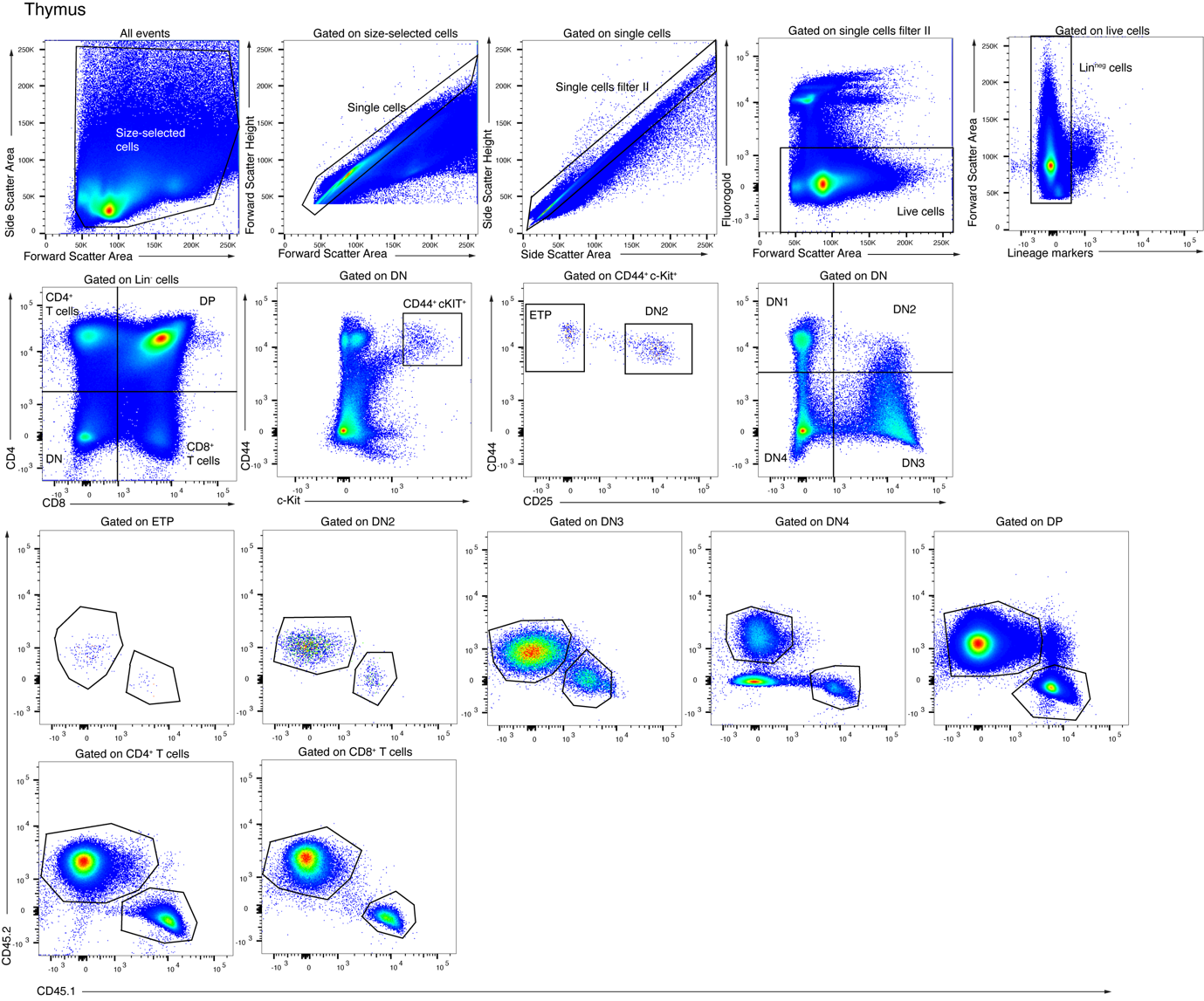
**

**Supplementary Figure 7. Gating strategy for the analysis of thymus 16 weeks after competitive foetal liver cell transplantation.**

Representative flow cytometry plots showing the identification of major progenitor populations and mature T cells in the thymus. DN1 to 4, populations 1 to 4 double negative for CD4 and CD8; DP, double positive for CD4 and CD8; ETP, early thymic progenitor cells. Lin, lineage markers for thymus analysis (B220, CD19, Mac-1, Gr-1, CD71, Ter-119).

The cell surface marker protein combinations for specific cell types are listed in Supplementary Table 2.

**Supplementary Table 1: Antibodies and antibody dilutions used for flow cytometry**

| Antibody | Source | Clone or catalogue | Dilution |
| --- | --- | --- | --- |
| anti-B220-A700 | WEHI | clone RA3-6B2 | 1:400 |
| anti-B220-APC | WEHI | clone RA3-6B2 | 1:400 |
| anti-B220-biotin | BD | 51-01122J | 1:200 |
| anti-CD127(IL7Ra)-biotin | BD | 555288 | 1:100 |
| anti-CD135(Flt3)-PE | BD | 553842 | 1:30 |
| anti-CD150-PE | Biolegend | 115904 | 1:100 |
| anti-CD19-A700 | WEHI | clone 1D3 | 1:400 |
| anti-CD19-biotin | WEHI | clone 1D3 | 1:400 |
| anti-CD19-FITC | WEHI | clone 1D3 | 1:400 |
| anti-CD19-PECy7 | BD | 552854 | 1:400 |
| anti-CD21-FITC | WEHI | clone 7G6 | 1:200 |
| anti-CD23-biotin | BD | 555709 | 1:200 |
| anti-CD24-PE | BD | 553263 | 1:200 |
| anti-CD25-PerCPCy5.5 | BD | 561112 | 1:200 |
| anti-CD34-FITC | eBioscience | 11-0341-82 | 1:30 |
| anti-CD4-A594 | WEHI | clone GK1.5 | 1:400 |
| anti-CD4-A700 | WEHI | clone GK1.5 | 1:400 |
| anti-CD4-PE | WEHI | clone GK1.5 | 1:400 |
| anti-CD43-biotin | BD | 553269 | 1:100 |
| anti-CD44-FITC | WEHI | clone 1M7 | 1:400 |
| anti-CD44-PE | BD | 553134 | 1:200 |
| anti-CD45.1-biotin | BD | 553774 | 1:100 |
| anti-CD45.1-BV650 | BD | 563754 | 1:200 |
| anti-CD45.1-PECy7 | Biolegend | 110730 | 1:100 |
| anti-CD45.2-A647 | WEHI | clone A20 | 1:400 |
| anti-CD45.2-A700 | WEHI | clone A21 | 1:400 |
| anti-CD45.2-PerCPCy5.5 | BD | 552950 | 1:100 |
| anti-CD48-FITC | eBioscience | 11-0481-85 | 1:100 |
| anti-CD71-FITC | WEHI | clone R17217.1.3 | 1:200 |
| anti-CD8-A700 | WEHI | clone 53.6.7 | 1:400 |
| anti-CD8-PECy7 | BD | 552877 | 1:400 |
| anti-c-Kit-APC | WEHI | clone ACK4 | 1:200 |
| anti-c-Kit-PerCPcy5.5 | Biolegend | 105824 | 1:200 |
| anti-Gr-1-A700 | WEHI | clone RB6-8C5 | 1:200 |
| anti-Gr-1-biotin | BD | 553125 | 1:200 |
| anti-IgD-PE | eBioscience | 12-5993-020 | 1:400 |
| anti-IgM-FITC | WEHI | clone 5.1 | 1:400 |
| anti-IgM-PE | WEHI | clone 5.1 | 1:400 |
| anti-Mac-1-A700 | WEHI | clone A7R34 | 1:200 |
| anti-Mac-1-biotin | BD | 553309 | 1:202 |
| anti-Mac-1-PE | WEHI | clone A7R34 | 1:200 |
| anti-Sca-1-A594 | WEHI | clone E13 | 1:200 |
| anti-Streptavidin-APC/Cy7 | BD | 554063 | 1:400 |
| anti-Streptavidin-BV650 | BD | 563855 | 1:400 |
| anti-Streptavidin-PerCPCy5.5 | BD | 551419 | 1:400 |
| anti-Streptavidin-Texas Red | BD | 562318 | 1:400 |
| anti-Ter-119-A700 | WEHI | clone TER-119 | 1:100 |
| anti-Ter-119-APC | WEHI | clone TER-119 | 1:100 |
| anti-Ter-119-biotin | BD | 553672 | 1:200 |

**Supplementary Table 2: Cell surface markers**

| **Cell type** | **Markers** |
| --- | --- |
| SLAM stem and progenitor cells (bone marrow; Figure 2; Figure 5D) | |
| LSK | Lin^neg^ Sca-1^+^ c-Kit^+^ |
| HSC | Lin^neg^ Sca-1^+^ c-Kit^+^ CD150^+^ CD48^neg^ |
| MPP | Lin^neg^ Sca-1^+^ c-Kit^+^ CD150^neg^ CD48^neg^ |
| HPC-1 | Lin^neg^ Sca-1^+^ c-Kit^+^ CD150^neg^ CD48^+^ |
| HPC-2 | Lin^neg^ Sca-1^+^ c-Kit^+^ CD150^+^ CD48^+^ |
| CD135/CD34 stem and progenitor cells (bone marrow; Figure 5C) | |
| LSK | Lin^neg^ Sca-1^+^ c-Kit^+^ |
| LT-HSC | Lin^neg^ Sca-1^+^ c-Kit^+^ CD135^neg^ CD34^neg^ |
| ST-HSC | Lin^neg^ Sca-1^+^ c-Kit^+^ CD135^neg^ CD34^+^ |
| MPP | Lin^neg^ Sca-1^+^ c-Kit^+^ CD135^+^ CD34^+^ |
| LMPP | Lin^neg^ Sca-1^+^ c-Kit^+^ CD135^hi^ CD34^+^ |
| Common lymphoid progenitor (bone marrow; Figure 5) | |
| CLP | Lin^neg^ Il7Rα^+^ c-Kit^int^ Sca-1^int^ |
| B-cell lineage (bone marrow and spleen; Figure 6A-E) | |
| Pre-Pro B (bone marrow) | B220^+^ CD19^neg^ c-Kit^neg^ CD43^+^ CD24^lo^ |
| Pro-B (bone marrow) | B220^+^ CD19^+^ c-Kit^neg^ CD43^+^ CD24^int^ |
| Pre-B (bone marrow) | B220^+^ CD19^+^ c-Kit^neg^ CD43^+^ CD24^hi^ |
| Immature B (bone marrow) | B220^+^ CD19^+^ c-Kit^neg^ sIgM^+^ sIgD^neg^ |
| Mature B (bone marrow) | B220^+^ CD19^+^ c-Kit^neg^ sIgM^+^ sIgD^+^ |
| T1 – immature (spleen) | B220^+^ CD19^+^ CD21^neg^ IgM^hi^ |
| T2 – immature (spleen) | B220^+^ CD19^+^ CD21^hi^ IgM^hi^ |
| Marginal zone B cells (spleen) | B220^+^ CD19^+^ CD21^hi^ CD23^neg^ |
| Mature B cells (spleen) | B220^+^ CD19^+^ CD21^int^ IgM^int/hi^ |
| T-cell lineage (thymus; Figure 6F-H)  B220-CD19-Mac1-Gr1-Ter119-  B220-CD19-Mac1-Gr1-Ter119- | |
| ETP (thymus) | Lin^neg^ c-Kit^+^ CD44^+^ CD25^neg^ |
| DN2 (thymus) | Lin^neg^ c-Kit^+^ CD44^+^ CD25^+^ |
| DN3 (thymus) | Lin^neg^ CD44^neg^ CD25^+^ |
| DN4 (thymus) | Lin^neg^ CD44^neg^ CD25^neg^ |
| Peripheral blood nucleated white blood cells (Figure 4) | |
| B cells | B220^+^ CD19^+^ |
| CD4 T cells | CD4^+^ CD8^neg^ |
| CD8 T cells | CD4^neg^ CD8^+^ |
| Myeloid | Gr-1^+^ Mac-1^+^ |
| Erythroid lineage (bone marrow, cells negative for Mac-1, Gr-1, CD45.2; Figure 3) | |
| Pro-erythroblast (I) | Ter-119^+^ CD44^hi^ forward scatter area^hi^ |
| Basophilic erythroblasts (II) | Ter-119^+^ CD44^int^ forward scatter area^hi^ |
| Polychromatic erythroblasts (III) | Ter-119^+^ CD44^int^ forward scatter area^int^ |
| Orthochromatic erythroblasts and reticulocytes (IV) | Ter-119^+^ CD44^int^ forward scatter area^lo^ |
| Mature erythrocyte (V) | Ter-119^+^ CD44^neg^ forward scatter area^lo^ |

Lineage markers ("Lin"): B220, CD4, CD8, CD19, Mac-1, Gr-1, Ter-119; Mac-1 is not used for foetal liver HSC Lin^neg^ sorts; CD4, CD8 are not used for thymus Lin^neg^ sort.

**References**

1. Adolfsson J, Borge OJ, Bryder D, Theilgaard-Mönch K, Astrand-Grundström I, Sitnicka E, et al., Upregulation of Flt3 expression within the bone marrow Lin(-)Sca1(+)c-kit(+) stem cell compartment is accompanied by loss of self-renewal capacity. Immunity (2001) 15:659-69.

2. Kondo M, Weissman IL, and Akashi K, Identification of clonogenic common lymphoid progenitors in mouse bone marrow. Cell (1997) 91:661-72.

3. Osawa M, Hanada K, Hamada H, and Nakauchi H, Long-term lymphohematopoietic reconstitution by a single CD34-low/negative hematopoietic stem cell. Science (1996) 273:242-5.

4. Kiel MJ, Yilmaz OH, Iwashita T, Yilmaz OH, Terhorst C, and Morrison SJ, SLAM family receptors distinguish hematopoietic stem and progenitor cells and reveal endothelial niches for stem cells. Cell (2005) 121:1109-21.

5. Oguro H, Ding L, and Morrison SJ, SLAM family markers resolve functionally distinct subpopulations of hematopoietic stem cells and multipotent progenitors. Cell Stem Cell (2013) 13:102-16.
